# Molecular detection of pathogenic Leptospira spp. in urban rodents from wet markets in northeast Malaysia

**DOI:** 10.5455/javar.2022.i593

**Published:** 2022-06-26

**Authors:** Intan Noor Aina Kamaruzaman, Muhamad Aiman Mohd Mokhtar, Hong Wei Ting, Yong Kai Yuan, Azim Wafiy Gulam Shah, Tan Wan Loong, Nurshahirah Shaharulnizim, Mohd Farhan Hanif Reduan, Fathin Faahimaah Abdul Hamid, Nur Amalina Noralidin, Nur Athirah Abdul Manaf, Che Wan Salma Che Wan Zalati, Loong Shih-Keng, Simon Clegg, Luqman Abu-Bakar

**Affiliations:** 1 Faculty of Veterinary Medicine, Universiti Malaysia Kelantan, Kota Bharu, Malaysia; 2 School of Life Sciences, University of Lincoln, Brayford Pool Lincoln, United Kingdom; 3 Tropical Infectious Diseases Research & Education Centre, Universiti Malaya, Kuala Lumpur, Malaysia

**Keywords:** Leptospires, Malaysia, molecular detection, PCR, rodents, wet market

## Abstract

**Objective:**

This short study describes the occurrence of pathogenic *Leptospira* spp. in two major wet markets in Kota Bharu, Kelantan, Malaysia.

**Materials and Methods:**

30 rodents (20 rats and 10 shrews) were caught in 2 wet markets, and a postmortem was performed to extract both kidneys. Molecular diagnosis via polymerase chain reaction (PCR) was conducted to detect leptospiral DNA using universal and pathogenic *Leptospira *primers, respectively.

**Results:**

The results showed that 20/28 (72%) rat samples were detected positive for *Leptospira *spp, and all shrews were negative. Further sequencing analysis identified *L. interrogans* and *L. borgpetersenii* as the most frequently *Leptospirosis* species from kidney samples.

**Conclusions:**

The presented study here sheds light on the presence of pathogenic leptospires harboring the rat population in both wet markets in Kelantan, which presents a great public health risk to wet market workers and visitors.

## Introduction

Leptospirosis is one of the global zoonotic diseases that primarily infects mammalian species, including humans. The disease accounts for more than 60,000 deaths worldwide [1] and causes major production losses in livestock in temperate countries. Leptospirosis is a notified disease in both the veterinary and human health sectors in Malaysia, and it is ranked third in terms of mortality after dengue and malaria [2]. Rodents are the principal carriers of leptospires, and the transmission of the disease from rats to high-density urban settings with poor sanitation is well-documented worldwide [3–5]. Leptospirosis is still a threat to people and animals because it has more than 250 pathogenic serovars, even though it is ignored and underreported in many countries [6].

Wet markets serve as the ideal place to disseminate various emerging diseases. In many low- and middle-income countries, the existence of these markets is part of the cultural heritage and traditions that are popular among the locals and tourists [7]. Various food products are sold daily, including live animals, freshly cut meats, vegetables, processed food products, etc. These edible products and the wet market environment are attractive to pests, such as rodents and insects, which devour the leftover food from the waste and continue to increase for years. In Malaysia, wet markets are present in all states, and several studies have linked leptospirosis in recent years [8–10]. Several factors, such as lack of hygiene, poor maintenance of the market, and inadequate hygienic preparation of meat and vegetables, may contribute to the spread of leptospirosis to human consumers.

The state of Kelantan (6.1254°N, 102.2381°E) is located in the northeastern part of Peninsular Malaysia. Kelantan has two major markets known among locals in the City Centre (Kota Bharu) (Wet Market A) and Pengkalan Chepa (Wet Market B). Both markets operate daily, with approximately 500–1,000 visitors [11]. Despite being the oldest markets in Kelantan, there are a limited number of studies on leptospirosis targeting rodents inhabiting both markets. So, this study was carried out to look for leptospires in rodents using the molecular method and to figure out what kind of leptospires were detected from the samples.

## Materials and Methods

### Ethical approval

Animal ethics approval was obtained via the intuitional animal care and use committee (IACUC) Universiti Malaysia Kelantan (ethics no.: UMK/FPV/ACUE/FYP/10/2021).

### Rat trapping

Rat (snap) traps were used to trap rodents at Wet Market A and B. All traps were set in the strategic area around the market at night, using fresh fish as bait. The trapping was set up from the end of October 2020 until early January 2021, a period spanning 10 weeks. The traps were inspected the following day. Trapped rodents were taken to the Animal Laboratory, Universiti Malaysia Kelantan, and transferred to a cage with unlimited access to food and water. Trapping procedures were repeated every 2 weeks until the desired number of animal samples was achieved (*n* = 40). For this study, all rodents were considered, regardless of their age and sex.

### Sample collection

All the laboratory procedures were conducted under BSL-2 facilities and practices. All animals fasted a day before the process. For the euthanasia procedure, the animal was euthanized using 2% (v/v) carbon dioxide in a closed air-tight chamber (Dira Resources, Malaysia). Postmortem was performed shortly after the animal died, and both kidneys were harvested immediately and kept at −80°C for downstream application.

### Molecular detection and characterization of Leptospira spp.

All kidney samples were subjected to DNA extraction before molecular detection and purification. Approximately 25 gm of kidney tissues were sectioned, weighed, and homogenized using a commercial DNA extraction kit (Macherey-Nagel, Dören, Germany), following the manufacturer’s instructions. Leptospiral DNA was amplified by polymerase chain reaction (PCR) using two sets of primers targeting 16S rRNA [12] and the LipL32 gene [13], detecting general *Leptospira* spp. and pathogenic *Leptospira* spp., respectively (Table 1). The condition cycle for both primers was set according to the following protocol suggested by previous studies, with slight modifications [12,13]. The initial denaturation was set at 95°C for 5 min, followed by the denaturation step at 95°C for 30 sec, annealing at 60°C (for both primers), the extension step at 72°C for 30 sec, and, lastly, the final extension at 72°C for 5 min. The DNA of *Leptospira interrogans* serovar (Canicola) was used as a positive control. PCR was set for 35 cycles, and the products were observed on a 1.5% (w/v) agarose gel stained with 1 μl Midori green and photographed under UV light. After visualization, positive samples were purified and submitted for sequencing. A phylogeny tree was modeled using Molecular Evolutionary Genetics Analysis version 11.0 (MEGA 11) (PSU, USA) to determine the relationship among the *Leptospira* spp. genera by applying the maximum-likelihood method [14].

## Results

### PCR detection of Leptospira spp.

**Figure 1.**
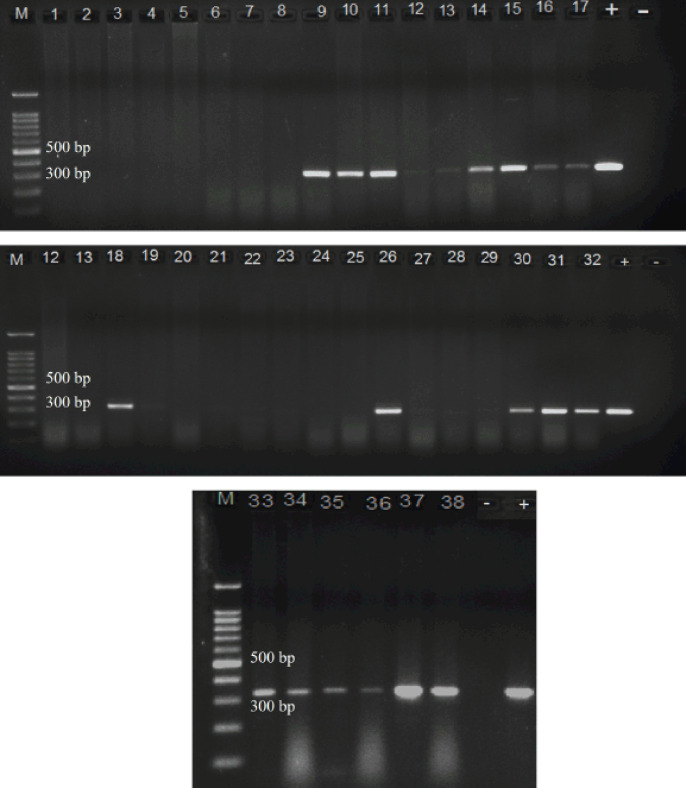
16S rRNA PCR amplification product bands of rodent kidney samples on 1.5% (w/v) agarose gels viewed under UV light. M: 1 kb marker, +/− (positive/negative) control. Positive control used in the study: *L. interrogans* serovar Copenhageni.

A total of 40 rodent kidneys consisting of 30 mixed-sex rats (*Rattus* spp.) and 10 shrews (*Crocidura*) were extracted. Two rat kidneys were found autolyzed during the process, making only 28 pairs of rodent kidney samples available for the research. The distribution of rodents belonging to the specific market is tabulated in Table 2. The DNA concentration was between 190 and 250 ng/ul and the purity was between 1.9 and 2.0, respectively. From the results, Wet Market A had slightly more positive animals (13/16) (59.1%) compared to Wet Market B (8/12) (50.0%). However, positive results were detected in the rat’s kidney samples and none in shrews. By PCR, 20 rat samples were detected positive using 16S rRNA PCR (71.4%) (Fig. 1) compared to LipL32 PCR (53.6%, 15 samples) (figure not shown). Overall, 16S rRNA PCR detected 52% of the positive *Leptospira* DNA in all animal samples, while LipL32 PCR detected 39% of the positive DNA. Except for five samples, all positive samples found with LipL32 PCR were also positive with 16S rRNA (Table 2).

**Table 1.** List of diagnostic leptospiral PCR primer pairs used in this study.

Primer	Target gene	Sequence (5’–3”)	Size (bp)	Specificity	Reference
LepF LepR	*16S rRNA*	5’-GGC GGC GCG TCT TAA ACA TG-3” 5’-TCC CCC CAT TGA GCA AGA TT-3”	330 bp	Universal *Leptospira* spp.	Mérien et al. [12]
Lep1132F Lep1132R	*LipL32*	5’-ATC TCC GTT GCA CTC TTT GC-3” 5’-ACC ATC ATC ATC ATC GTC CA-3”	756 bp	Pathogenic *Leptospira* spp.	Ibrahim et al. [13]

**Table 2.** PCR detection results of *Leptospira* spp. in animal samples in this study.

Animal	No. of samples	No. of positive rodents by PCR with %		No. of positive rodents in selected wet marketd	
		16S rRNAa	LipL32b	Wet Market A	Wet Market B
Rat (*Rattus* spp.)	28	20 (71.4%)	15 (53.6%)	13/16	8/12
Shrew (Genus *Crocidura*)	10	0 (0%)	0 (0%)	0/6	0/4
Total		20/38	15/38	13/22	8/16
**PCR positivity (%)**		**52%**	**39%**	**59.1%**	**50.0%**

^a^Positive samples detected in 16S rRNA were also found positive in LipL32 PCR.

^b^Positive samples detected in LipL32 PCR were also found positive in 16S rRNA PCR, except for five samples.

^c^Original kidney samples was 40; however, two kidneys samples were autolyzed, thus not available.

^d^The number of positive animals over the number of captured rodents in the selected market.

### Phylogenetic analysis

**Figure 2.**
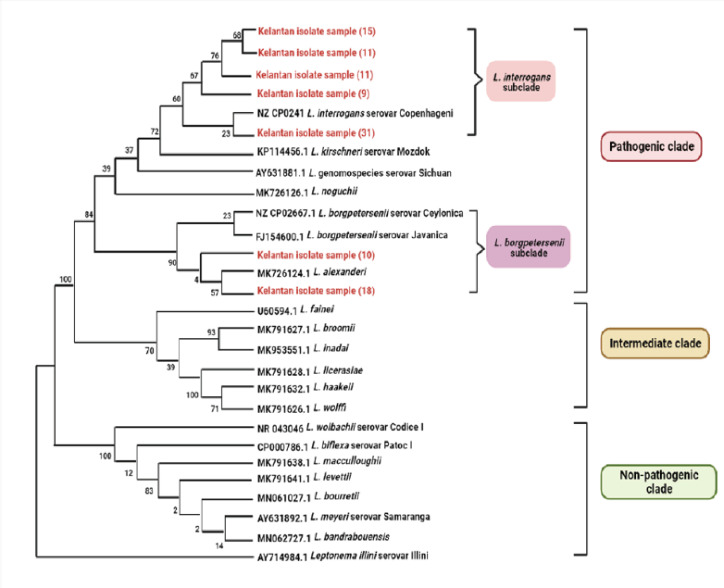
The phylogenetic analysis of the amino acid sequence of 16S rRNA across various pathogenic *Leptospira* genomospecies and serovars by maximum likelihood method based on ~330 aligned based pairs constructed using MEGA11 [14]. Bootstrapping was performed 1,000 times, and all positions containing gaps and missing data were removed. Positive samples in the study are highlighted.

Out of 20 positive samples, only 8 were successfully submitted for full sequencing analysis. Reamplifying the remaining samples generated weak PCR products and yielded poor sequence results. A phylogenetic model was constructed based on 16S rRNA nucleotide analysis across *Leptospira* pathogenicity groups (pathogenic, intermediate, and nonpathogenic clades) consisting of reference genomospecies (Fig. 2). From the sequence analysis, it was found that the majority of *Leptospira* spp. detected in this study are highly similar to *L. interrogans* (100%), and two samples are identical to *L. borgpetersenii* (98%), respectively. Five previously detected positive samples using 16S rRNA PCR, but not in LipL32 PCR, were considered to belong to either the nonpathogenic or intermediate *Leptospira* group.

## Discussion

This study showed pathogenic *Leptospira* spp. in urban rodents captured at two major wet markets in Kota Bharu, Kelantan. The literature search shows this is the first study targeting rodents at both wet markets. Recently, Kelantan showed an exponential of human leptospirosis cases [15], which could be attributed to the exposure to the bacteria via rodent transmission to the human setting. The wet market is ideal as their breeding ground and could potentially become an epicenter for leptospirosis spreading, which will be an extremely high risk to wet market workers and visitors. A previous study by Rahman et al. [8] showed that wet market merchants in Kota Bharu, Kelantan, were directly exposed to pathogenic *Leptospira* spp., with a high seroprevalence rate. Interestingly, a similar study in wet markets in another Malaysian state (Selangor) also reported a high *Leptospira* seroprevalence rate among wet market merchants and food handlers [9]. These results back up the idea that these people are always at risk of occupational hazards, which is bad for public health.

In this study, both markets showed significant positive leptospires occurrence in rats. Surprisingly, none of the captured shrews were found to be positive. Although most shrew species are carriers of specific *Leptospira* serovars, the occurrence of pathogenic *Leptospira* spp. is not common in the shrew population. However, interaction with rats may lead to pathogenic species transmission to the shrew population [16,17]. More studies are warranted to determine the circulating Leptospira serovars within shrew species and to elucidate if this animal can harbor pathogenic *Leptospira* serovars as both maintenance and bystander infections.

From the PCR results (Table 2), it is suggested that 16s rRNA has a reasonable detection rate (20/38) compared to LipL32 (15/38) PCR; thus, it is still considered reliable for diagnostic work. However, LipL32 has better specificity to detect all pathogenic *Leptospira* spp. as the LipL32 gene is present only in the pathogenic species [18,19], which is a choice for many leptospiral detection studies in humans, animals, and the environment [20–22]. In this study, all positive samples (20/38) detected via 16S rRNA were also detected as positive in LipL32 PCR (15/38) except for five samples (Table 2). These samples were repeated for both PCR cycles and yielded similar results. However, we could still not submit these products for sequence due to poor field after purification. Hence, we consider that these five samples belong to the nonpathogenic/intermediate clade. We think that both PCRs are very sensitive and can be used for quick detection because of this.

From the phylogenetic analysis, all samples belong to the two major pathogenic subclusters: *L. interrogans* (six samples) and *L. borgpetersenii* (two samples). Interestingly, all samples showed 100% identity to *L. interrogans* serovar Copenhageni, and two samples had 98% identity to L. borgpetersenii serovar Javanica, respectively. These findings are similar to a recent study by Bahtiar Affendy et al. [10], who reported both strains isolated from small mammals in wet markets. Although both strains are human pathogens, L. interrogans is considered highly pathogenic because it can survive outside the host and remain viable in the environment for a long time [23], whereas *L. borgpetersenii *is a host-restricted pathogen with limited survival capability [24]. Leptospirosis caused by both strains has been reported in human and animal hosts worldwide in recent years [25–29]. Contact with rodent carriers enhances the transmission of these bacteria to vulnerable hosts, and it is speculated that rats are chronically infected for a lifetime and are able to pass the bacteria to their offspring via vertical routes, such as *in utero* and milk transmission [30].

Several limitations were noted and can be planned for improvement. For example, a serodiagnosis study using samples from animals and wet market workers via Microscopic Agglutination Test (MAT) may be ideal for determining the predominant circulating leptospires in the study area that may be linked to future disease transmission. Lastly, wet market workers and visitors in Kelantan should be asked about their knowledge, attitudes, and practices to see how much they know about leptospirosis and how it spreads.

## Conclusion

In conclusion, we successfully proved that rats in two significant markets in Kelantan carry more pathogenic leptospires and potentially pose a public health risk to wet market workers and visitors. To the best of our knowledge, this is the first study targeting rodent leptospirosis in both wet markets in local cities in Kelantan. The study provides essential information on the presence of circulating leptospires in the rat population to flag the local council/health authorities to promote an awareness campaign among Kota Bharu and Pengkalan Chepa residents and to carry out aggressive rodent control programs as an effective preventive measure to contain the transmission of leptospirosis to humans. Further studies are warranted to investigate potential leptospires reservoirs via bacterial isolation from small mammals and environmental samples from the wet markets.

## List of abbreviations

PCR: Polymerase chain reaction; MEGA 11: Molecular Evolutionary Genetics Analysis version 11.0.
